# Concomitant Administration of Ozanimod and Serotonergic Antidepressants in Patients With Ulcerative Colitis or Relapsing Multiple Sclerosis

**DOI:** 10.1093/ibd/izae136

**Published:** 2024-07-17

**Authors:** Miguel Regueiro, Britta Siegmund, Sara Horst, Ryan Moslin, Lorna Charles, AnnKatrin Petersen, Daniel Tatosian, Hsiuanlin Wu, Garrett Lawlor, Monika Fischer, Geert D’Haens, Jean-Frederic Colombel

**Affiliations:** Cleveland Clinic, Cleveland, OH, USA; Department of Gastroenterology, Infectious Diseases, and Rheumatology, Charité–Universitätsmedizin Berlin, corporate member of Freie Universität Berlin and Humboldt-Universität zu Berlin, Berlin, Germany; Vanderbilt University Medical Center, Nashville, TN, USA; Bristol Myers Squibb, Princeton, NJ, USA; Bristol Myers Squibb, Princeton, NJ, USA; Bristol Myers Squibb, Princeton, NJ, USA; Bristol Myers Squibb, Princeton, NJ, USA; Bristol Myers Squibb, Princeton, NJ, USA; Bristol Myers Squibb, Princeton, NJ, USA; Indiana University School of Medicine, Indianapolis, IN, USA; Academic Medical Center Amsterdam, Amsterdam, Netherlands; Icahn School of Medicine at Mount Sinai, New York, NY, USA

**Keywords:** ulcerative colitis, ozanimod, serotonergic antidepressants, serotonin accumulation, safety

## Abstract

**Background:**

Ozanimod, approved for the treatment of moderately to severely active ulcerative colitis (UC) and relapsing multiple sclerosis (RMS), is a weak in vitro monoamine oxidase B (MAO-B) inhibitor. MAO-B inhibitors can cause serotonin accumulation with concomitant use of selective serotonin reuptake inhibitors (SSRIs) or serotonin and norepinephrine reuptake inhibitors (SNRIs). We evaluated the incidence of treatment-emergent adverse events (TEAEs) potentially associated with serotonin accumulation during ozanimod and concomitant SSRI/SNRI use in this post hoc analysis of pooled UC studies and the open-label extension RMS DAYBREAK.

**Methods:**

Data for ozanimod 0.92 mg from pooled UC studies (n =* *1158; cutoff: January 10, 2022) and RMS DAYBREAK (n = 2257; cutoff: February 1, 2022) were analyzed. Concomitant SSRI/SNRI use was allowed in the UC (n = 67) and RMS (n* = *274) studies. A narrow Medical Dictionary for Regulatory Activities search (“serotonin syndrome,” “neuroleptic malignant syndrome,” and “malignant hyperthermia”) and a broad search including terms potentially associated with serotonin accumulation were conducted. The percentages of patients with TEAEs in both searches were analyzed by concomitant SSRI/SNRI use when the TEAE occurred.

**Results:**

No patients had TEAEs matching the narrow search criteria. No differences were observed in the percentages of patients with ≥1 TEAE matching the broad search regardless of SSRI/SNRI use in UC (with: 25.4% [n = 17 of 67]; without: 15.0% [n = 164 of 1091]) and RMS (with: 12.4% [n = 34 of 274]; without: 15.6% [n = 310 of 1982]) studies.

**Conclusions:**

No evidence of increased TEAEs potentially associated with serotonin accumulation was observed with concurrent use of ozanimod and SSRIs/SNRIs.

**Clinical trial registration:**

NCT01647516, NCT02531126, NCT02435992, NCT02576717

## Graphical Abstract

**Graphical Abstract:**
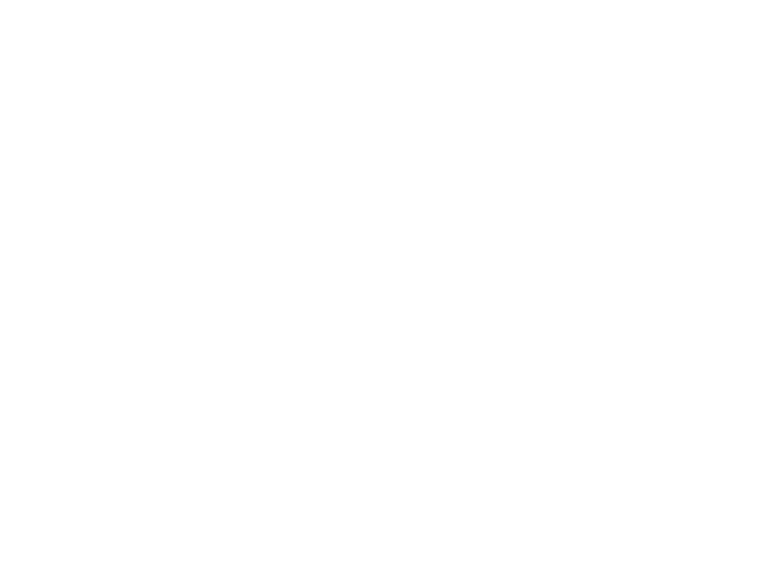

**Key Messages**

- What is already known?
  ◦ Ozanimod is a weak monoamine oxidase B inhibitor in vitro. Monoamine oxidase B inhibitors and selective serotonin reuptake inhibitors/serotonin and norepinephrine reuptake inhibitors used concomitantly can cause serotonin accumulation.
- What is new here?
  ◦ No increased risk of adverse events associated with potential serotonin accumulation, regardless of concomitant use of ozanimod and a selective serotonin reuptake inhibitor/serotonin and norepinephrine reuptake inhibitor, occurred in patients with ulcerative colitis or relapsing multiple sclerosis.
- How can this study help patient care?
  ◦ Together with the prescribing information, our findings help clinicians assess the risk-benefit of initiating ozanimod in patients on concomitant serotonergic antidepressants.

## Introduction

Mental health comorbidities (eg, anxiety, depression) are common in patients with chronic diseases, such as inflammatory bowel disease (IBD) and multiple sclerosis (MS).^1-3^ Patients with IBD have at least twice the risk of developing mental health comorbidities compared with the overall population, and up to 54% of patients with MS experience anxiety or depression.^3-6^ Therefore, those with ulcerative colitis (UC) or MS may be on selective serotonin reuptake inhibitors (SSRIs) or serotonin and norepinephrine reuptake inhibitors (SNRIs), which are first-line pharmacotherapeutic treatment options for patients with anxiety and depression.^7,8^

Although SSRIs and SNRIs are generally well tolerated, patients should be monitored for drug–drug interactions, especially with the coadministration of SSRIs/SNRIs and other serotonergic agents, such as monoamine oxidase (MAO) inhibitors.^8-12^ Inhibition of either of the 2 MAO isoforms, MAO-A and MAO-B, can block the metabolism of neurotransmitters (eg, serotonin), leading to the accumulation of neurotransmitters in the central nervous system.^12,13^ The coadministration of treatments that increase serotonin, including an MAO inhibitor with an SSRI/SNRI, could cause serotonin to accumulate, potentially leading to adverse reactions.^12,13^ The most severe—but rare—potential adverse reactions of serotonin accumulation are hypertensive crisis and serotonin syndrome.^9^

Ozanimod, a selective sphingosine 1-phosphate receptor 1 and 5 modulator, is approved for the treatment of moderately to severely active UC and relapsing multiple sclerosis (RMS).^14-16^ Ozanimod prevents lymphocyte trafficking to inflamed tissues in the gastrointestinal tract and central nervous system to reduce inflammation in patients with UC and RMS, respectively.^16-18^ Ozanimod is metabolized into 2 major active metabolites, CC112273 and CC1084037, that are weak inhibitors of MAO-B in vitro (half-maximal inhibitory concentration [IC_50_] greatly exceeds therapeutic concentrations).^19^ The potential for clinical MAO interaction was initially explored in a phase 1, multiple-dose pharmacokinetic study of ozanimod and pseudoephedrine, a sympathomimetic agent.^19^ Coadministration of an MAO inhibitor and sympathomimetic can result in blood pressure elevations that could potentially lead to hypertensive crisis due to excess neurotransmitter accumulation.^19^ Results of this study demonstrated that the daily coadministration of ozanimod 1.84 mg, which is double the recommended dose, with a single dose of pseudoephedrine 60 mg did not cause clinically significant differences in blood pressure compared with pseudoephedrine alone.^19^ Ozanimod was also found to not inhibit human platelet MAO-B activity in these subjects.^19^

A previously published analysis of data from the open-label extension (OLE) DAYBREAK RMS study showed that concomitant SSRI/SNRI and ozanimod use was not associated with an increase in treatment-emergent adverse events (TEAEs) potentially associated with serotonin accumulation.^20^ This post hoc analysis evaluated previously published data in patients with RMS from the OLE DAYBREAK study^20^ in addition to data from the pooled UC studies to determine the incidences of TEAEs potentially related to serotonin accumulation, including hypertension, during the concomitant use of ozanimod with SSRIs or SNRIs.

## Methods

**Figure 1.**
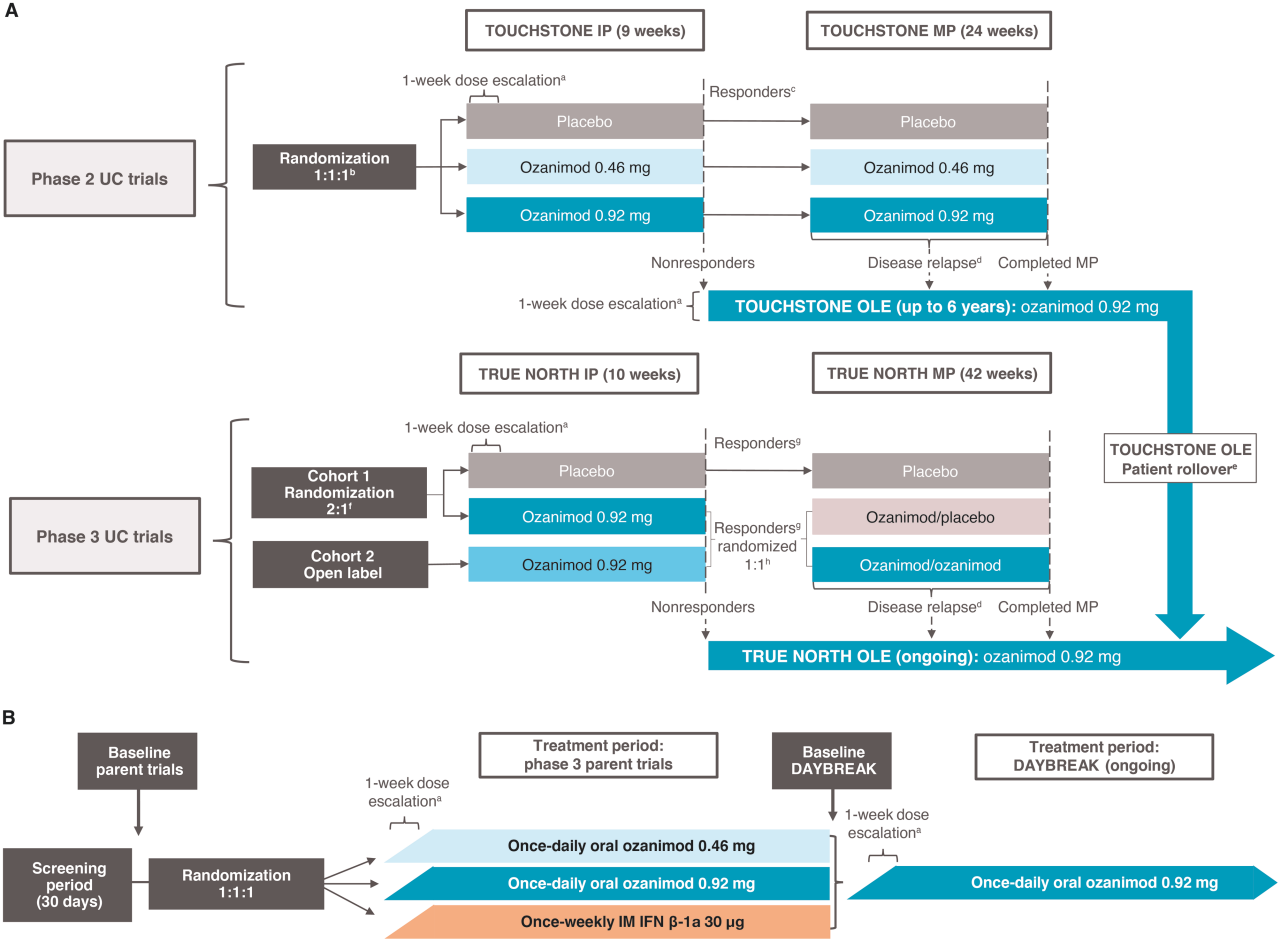
Study design of the (A) pooled ulcerative colitis (UC) studies and (B) relapsing multiple sclerosis (RMS) DAYBREAK study.^21-23,25^^a^Patients randomized to ozanimod received ozanimod 0.23 mg (equivalent to ozanimod hydrochloride 0.25 mg) on days 1 to 4, ozanimod 0.46 mg (equivalent to ozanimod hydrochloride 0.5 mg) on days 5 to 7, and their assigned dose of ozanimod 0.46 or ozanimod 0.92 mg (equivalent to ozanimod HCl 1 mg) starting on day 8. ^b^Patients stratified by prior tumor necrosis factor (TNF) inhibitor exposure (yes/no). ^c^Reduction in 4-component Mayo score of ≥3 points and ≥30%, and reduction in rectal bleeding subscore (RBS) of ≥1 point or absolute RBS of ≤1 point. ^d^Disease relapse was defined as partial Mayo score increase ≥2 points vs the partial Mayo score at maintenance period (MP) entry and absolute partial Mayo score ≥4 points, endoscopic subscore of ≥2 points, and exclusion of other causes of an increase in disease activity unrelated to underlying UC. ^e^In 2019, the sponsor ended the open-label extension (OLE) and rolled over all active patients who consented to a phase 3 program (all had completed at least OLE week 200). ^f^Patients stratified by prior TNF inhibitor exposure (yes/no) and corticosteroid use (yes/no) at screening. ^g^Reduction in 3-component Mayo score of ≥2 points and ≥35%, or reduction in 4-component Mayo score of ≥3 points and ≥30%, and reduction in RBS of ≥1 point or absolute RBS of ≤1 point. ^h^Responders assigned to ozanimod (cohorts 1 and 2) in the induction period (IP) were rerandomized 1:1 to ozanimod or placebo in a double-blind manner when entering the MP; adult patients in clinical response at week 10 of the IP who were randomized to placebo (cohort 1) continued to receive placebo in the MP in a double-blind manner. ^i^Patients who entered from a blinded parent study or treatment period (True North cohort 1) underwent the 1-week dose escalation for ozanimod. IFN, interferon; IM, intramuscular.

This post hoc analysis included patients receiving ozanimod 0.92 mg with moderately to severely active UC from the pooled UC studies—phase 2 TOUCHSTONE study (NCT01647516),^21^ phase 2 TOUCHSTONE OLE (NCT02531126),^22^ phase 3 True North study (NCT02435992),^23^ and phase 3 True North OLE (NCT02576717) (Figure 1A)^24^ and patients with RMS receiving ozanimod 0.92 mg who entered the DAYBREAK OLE study^25^ from the phase 3 SUNBEAM^26^ and RADIANCE^27^ studies (Figure 1B). Brief overviews on the study methodologies are described in Study Design, but the TOUCHSTONE,^21^ TOUCHSTONE OLE,^22^ True North,^23^ True North OLE,^24^ SUNBEAM,^26^ RADIANCE,^27^ and DAYBREAK^25^ studies have been previously described.

### Ethical Considerations

This analysis adhered to the Good Clinical Practices guidelines and the ethical principles outlined in the Declaration of Helsinki. The protocol was approved by the institutional review board at each center. All patients provided written informed consent, and this study was sponsored by Bristol Myers Squibb.

### Study Design

#### Pooled UC studies

TOUCHSTONE (NCT01647516) was a double-blind, placebo-controlled, 32-week phase 2 study (9-week induction period [IP] followed by a 24-week maintenance period [MP]) that assessed the efficacy and safety of ozanimod 0.92 mg or ozanimod 0.46 mg/d (equivalent to ozanimod hydrochloride 0.5 mg) in patients with UC.^21^ Patients from TOUCHSTONE were eligible to enroll in the OLE (NCT02531126) to receive ozanimod 0.92 mg/d if they were week 8 IP ozanimod nonresponders, had disease relapse during the MP, or completed the MP and wanted to continue therapy.^22^ The TOUCHSTONE OLE reported the long-term efficacy and safety of ozanimod 0.92 mg/d for about 4 years (≥200 weeks of treatment) until the study ended.^22^ Eligible patients remaining at the end of the study could continue therapy and roll over into the phase 3 True North OLE.^22^

True North (NCT02435992) was a multicenter, randomized, double-blind, placebo-controlled, 52-week phase 3 study (IP: 10 weeks; MP: 42 weeks) that evaluated the efficacy and safety of ozanimod 0.92 mg/d in patients with UC.^23^ Patients from True North could enter the OLE (NCT02531126) if they did not respond to ozanimod at week 10, had disease relapse during the MP, or completed week 52 MP and wanted to continue therapy.^23^ The True North OLE is an ongoing, open-label, multicenter extension study that aims to evaluate the long-term safety and efficacy of ozanimod 0.92 mg/d in patients with UC. The True North OLE included eligible patients from True North and those who completed ≥1 year of the TOUCHSTONE OLE. Patients who entered from a blinded treatment period underwent the 1-week ozanimod dose-escalation period. In this analysis, the data cutoff for the True North OLE was January 10, 2022.

#### RMS OLE: DAYBREAK

SUNBEAM (NCT02294058; duration: ≥12 months) and RADIANCE (NCT02047734; duration: 24 months) were multicenter, randomized, double-blind, double-dummy, active-controlled phase 3 studies that assessed the efficacy and safety of ozanimod 0.92 mg or ozanimod 0.46 mg/d in patients with RMS.^26,27^ Those who completed SUNBEAM or RADIANCE were able to enroll in the ongoing, single-arm, multicenter DAYBREAK OLE study (NCT02576717), which aimed to evaluate the long-term safety and efficacy of ozanimod 0.92 mg/d in patients with RMS.^25^ The DAYBREAK data cutoff for this analysis was February 1, 2022.^20^

### Patients

In the pooled UC studies, patients were 18 to 75 years of age with moderately to severely active UC, which was characterized as having a Mayo score of 6 to 12 and endoscopic subscore ≥2 in TOUCHSTONE and a total Mayo score of 6 to 12 (endoscopy subscore ≥1, rectal bleeding subscore ≥1, and stool frequency subscore ≥1) in True North.^21,23^ Patients who entered the RMS DAYBREAK OLE from SUNBEAM and RADIANCE were 18 to 55 years of age with RMS, had MS-consistent brain lesions as seen on magnetic resonance imaging, and had an Expanded Disability Status Scale score of 0 to 5.^25-27^ All pooled UC studies and the RMS DAYBREAK study permitted concomitant use of SSRIs or SNRIs. This analysis included all patients receiving ozanimod with concomitant use of SSRIs or SNRIs during the pooled UC and RMS DAYBREAK studies.

### Outcomes

A primary narrow Medical Dictionary for Regulatory Activities (MedDRA; version 25.1 in UC and version 24.0 in RMS) search was conducted using the following preferred terms: “serotonin syndrome,” “neuroleptic malignant syndrome,” and “malignant hyperthermia” (due to their overlapping symptoms).^20^ Patients were counted once per system organ class or preferred term for multiple occurrences. This search was supplemented with a secondary, broader MedDRA search based on a standardized MedDRA query for neuroleptic malignant syndrome (substantial overlap of potential symptoms with serotonin accumulation) to look for clustering of TEAEs that might infer risk of underlying serotonin syndrome. Incidences of TEAEs from the broad MedDRA search were captured >1 day from the start date of concomitant treatment with SSRIs or SNRIs and ozanimod; >1 day was used to be reasonably sure that the TEAE started after initiation of medication.^20^ The percentages of patients with TEAEs matching terms in the narrow and broad MedDRA searches were evaluated. For each preferred term, the patients were analyzed by concomitant SSRI or SNRI use at the time when the TEAE occurred.

### Statistical Analysis

The safety population included all patients who received ≥1 dose of ozanimod. Safety data were analyzed using descriptive summaries, and categorical data were summarized using frequency counts and percentages. The percentages of patients with ≥1 TEAE matching the terms from each MedDRA search were determined and stratified by concomitant SSRI or SNRI use and compared using the Fisher exact test and chi-square test for the UC and RMS analyses, respectively.^20^ All statistical comparisons were considered hypothesis generating, and *P* values were nominal. The incidences of TEAEs that were potentially related to serotonin accumulation were reported.

## Results

### Study Population

This analysis included 1158 patients receiving ozanimod in the pooled UC studies. Of the 2257 patients evaluated in the RMS DAYBREAK study, 2256 receiving ozanimod were included in this analysis^20^; 1 patient did not receive any doses of ozanimod. The patient demographic and baseline characteristics were based on the 2257 patients with RMS who were evaluated.^20^ At data cutoff, the mean exposure to ozanimod was 28.4 ± 23.3 months, and the total exposure of ozanimod was 2714.9 patient-years in the pooled UC studies; in the RMS DAYBREAK study, the mean exposure to ozanimod was 56.1 ± 15.3 months, and the total exposure of ozanimod was 10 540 patient-years.

This analysis included 67 (5.8%) and 274 (12.1%) patients who received concomitant SSRI or SNRI in the pooled UC studies and RMS DAYBREAK study, respectively.^20^ These patients were exposed to ≥1 SSRI or SNRI for a mean of 27.7 ± 22.6 and 37.0 ± 24.6 months with a total concomitant SSRI or SNRI and ozanimod exposure of 154.8 and 845.0 patient-years during the pooled UC studies and the RMS DAYBREAK study, respectively.^20^

Overall, patient demographic and baseline characteristics were similar in the pooled UC and RMS DAYBREAK studies (Table 1). However, a higher proportion of patients in the pooled UC studies were male (59.4% [n = 668 of 1158]) compared with RMS DAYBREAK (33.5% [n = 755 of 2257]).^20^ In addition, more patients in the pooled UC studies had depression (5.4% vs 4.8%) and anxiety (4.5% vs 1.7%) compared with those in RMS DAYBREAK, and slightly more patients received a concomitant SSRI or SNRI in RMS DAYBREAK (12.1%) compared with the pooled UC studies (5.8%).^20^

**Table 1.** Patient demographic and clinical characteristics in the pooled UC studies and the open-label RMS DAYBREAK study.^20^

Characteristic	Pooled UC studies	RMS DAYBREAK
	Ozanimod 0.92 mg (N = 1158)	Ozanimod 0.92 mg (N = 2257)^a^
**Age, y**	41.6 ± 13.3	37.3 ± 9.1
**Sex**		
Female	470 (40.6)	1502 (66.5)
Male	688 (59.4)	755 (33.5)
**Race**		
Asian	68 (5.9)	1 (<0.1)
Black or African American	31 (2.7)	9 (0.4)
White	1036 (89.5)	2244 (99.4)
Other	22 (1.9)	3 (0.1)
**BMI, kg/m** ^**2**^		
Mean (SD)	25.4 ± 5.4^b^	24.2 ± 4.7^c^
**BMI category**		
<30 kg/m^2^	962 (83.1)	2022 (89.6)^c^
**Psychological disorders** ^d^		
Depression	62 (5.4)	108 (4.8)
Anxiety	52 (4.5)	39 (1.7)
Anxiety disorder	5 (0.4)	9 (0.4)
Generalized anxiety disorder	2 (0.2)	1 (<0.1)
Persistent depressive disorder	2 (0.2)	0
Adjustment disorder with anxiety	1 (<0.1)	0
Depressed mood	1 (<0.1)	8 (0.4)
Major depression	1 (<0.1)	1 (<0.1)
Mixed anxiety and depressive disorder	1 (<0.1)	0
Social anxiety disorder	1 (<0.1)	0
Anxiety disorder due to a general medical condition	0	1 (<0.1)
**Concomitant SSRI/SNRI use**	67 (5.8)	274 (12.1)

Values are mean ± SD or n (%).

Abbreviations: BMI, body mass index; RMS, relapsing multiple sclerosis; SNRI, serotonin and norepinephrine reuptake inhibitor; SSRI, selective serotonin reuptake inhibitor; UC, ulcerative colitis.

^a^1 patient did not receive the study drug.

^b^n* = *1155.

^c^At parent study baseline.

^d^Not all are listed.

### Potential Serotonin-Related TEAEs Based on the Narrow Search Criteria

No patients had TEAEs matching the narrow search criteria of “serotonin syndrome,” “neuroleptic malignant syndrome,” or “malignant hyperthermia” in the pooled UC studies or the RMS DAYBREAK study.^20^

### Potential Serotonin-Related TEAEs Based on the Broad Search Criteria

The proportions of patients with ≥1 TEAE matching the broad criteria in the secondary search in the pooled UC studies were 25.4% (n = 17 of 67) for concomitant SSRI or SNRI use vs 15.0% (n = 164 of 1091) for no concomitant SSRI or SNRI use; no patients had >1 TEAE or a cluster of TEAEs compatible with serotonin accumulation. The difference in the proportions of patients with ≥1 TEAE (10.3%; 95% confidence interval, −0.3% to −21.0%) was nominally significant (*P* = .0355). In the RMS DAYBREAK study, 12.4% (n = 34 of 274; concomitant SSRI or SNRI) vs 15.6% (n = 310 of 1982; no concomitant SSRI or SNRI) matched, and the difference in the proportions of patients with ≥1 TEAE (−3.2%; 95% confidence interval, −7.5% to 1.0%) was not nominally significant (*P* = .1630).^20^

In general, the incidences of potential serotonin-related TEAEs were similar between patients who did and did not receive concomitant SSRIs or SNRIs in the pooled UC studies and RMS DAYBREAK study (Table 2). Notably, none of the 17 patients with concomitant SSRI or SNRI use in the pooled UC studies reported >1 potential serotonin-related TEAE. A few patients with concomitant SSRI or SNRI use in the RMS DAYBREAK study experienced >1 potential serotonin-related TEAE. The most common potential serotonin-related TEAEs reported in the pooled UC studies were nausea (6.0%) and hypertension (6.0%) in patients with concomitant SSRI or SNRI use and hypertension (5.4%) in patients without concomitant SSRI or SNRI use. In the RMS DAYBREAK study, hypertension was the most common TEAE, regardless of concomitant SSRI or SNRI use (concomitant SSRI or SNRI: 4.7%; no concomitant SSRI or SNRI: 8.5%).

**Table 2.** TEAEs potentially related to serotonin in the broad MedDRA search by SSRI/SNRI use in the pooled UC studies and RMS DAYBREAK study.^20^

	Pooled UC studies		RMS DAYBREAK	
	Concomitant SSRI/SNRI use (n = 67)	No concomitant SSRI/SNRI use (n = 1091)^a^	Concomitant SSRI/SNRI use (n = 274)^a^	No concomitant SSRI/SNRI use (n = 1982)^a^
**Patients with ≥1 potential serotonin-related TEAE**	17 (25.4)^b^	164 (15.0)	34 (12.4)^b^	310 (15.6)
**TEAE**				
Nausea	4 (6.0)	48 (4.4)	2 (0.7)	20 (1.0)
Hypertension	4 (6.0)	59 (5.4)	13 (4.7)	168 (8.5)
Pyrexia	3 (4.5)	41 (3.8)	4 (1.5)	36 (1.8)
Insomnia	2 (3.0)	13 (1.2)	12 (4.4)	56 (2.8)
Tremor	2 (3.0)	0	0	7 (0.4)
Blood pressure increased	1 (1.5)	4 (0.4)	2 (0.7)	23 (1.2)
Presyncope	1 (1.5)	0	0	2 (0.1)
Tachycardia	0	5 (0.5)	1 (0.4)	5 (0.3)
Hyperthermia	0	4 (0.4)	0	12 (0.6)
Hypotension	0	2 (0.2)	0	7 (0.4)
White blood cell count increased	0	2 (0.2)	0	2 (0.1)
Hyperpyrexia	0	2 (0.2)	0	0
Aggression	0	1 (0.1)	0	0
Agitation	0	1 (0.1)	0	0
Blood pressure decreased	0	1 (0.1)	0	1 (<0.1)
Body temperature increased	0	1 (0.1)	1 (0.4)	8 (0.4)
Autonomic nervous system imbalance	0	1 (0.1)	0	1 (<0.1)
Parkinsonism	0	1 (0.1)	0	0
Leukocytosis	0	1 (0.1)	0	2 (0.1)
Dystonia	0	0	2 (0.7)	0
Loss of consciousness	0	0	1 (0.4)	0
Heart rate increased	0	0	0	3 (0.2)
Blood pressure fluctuation	0	0	0	3 (0.2)
Myoclonus	0	0	0	2 (0.1)
Hypertonia	0	0	0	1 (<0.1)
Labile hypertension	0	0	0	1 (<0.1)
Muscle rigidity	0	0	0	1 (<0.1)
Disorientation	0	0	0	1 (<0.1)

Values are n (%).

Abbreviations: MedDRA, Medical Dictionary for Regulatory Activities; RMS, relapsing multiple sclerosis; SNRI, serotonin and norepinephrine reuptake inhibitor; SSRI, selective serotonin reuptake inhibitor; TEAE, treatment-emergent adverse event; UC, ulcerative colitis.

^a^Patients may have reported ≥1 TEAE.

^b^Only TEAEs that occurred while patients were on ozanimod and an SSRI/SNRI were included.

## Discussion

Given the increasing prevalence of mental health comorbidities in patients with IBD and MS, it is important to determine whether any TEAEs are associated with the concomitant use of ozanimod and an SSRI or SNRI.^3,4,13,19^ Results of this post hoc analysis of the pooled UC and RMS DAYBREAK studies did not provide evidence of an increased risk of TEAEs associated with potential serotonin accumulation, regardless of concomitant use of ozanimod and an SSRI or SNRI. Although the difference in the proportions of patients with ≥1 potential serotonin-related TEAE in the concomitant SSRI or SNRI use group vs no concomitant SSRI or SNRI use group was nominally significant in the pooled UC studies, this difference was likely due to the small number of patients in the concomitant SSRI or SNRI use group. Our findings support the absence of clinically meaningful ozanimod MAO-B inhibition in vivo.

A phase 1, multiple-dose pharmacokinetic study of ozanimod and pseudoephedrine, a sympathomimetic agent, evaluated the potential interaction between the 2 treatments, as coadministration of an MAO inhibitor and sympathomimetic can result in blood pressure elevations that potentially lead to hypertensive crisis due to excess neurotransmitter accumulation.^19^ Results of that study indicated that concomitant use of ozanimod and pseudoephedrine did not cause significant elevations in blood pressure, demonstrating the lack of in vivo MAO-B inhibition by ozanimod, even at high doses of 1.84 mg/d.^19^ Results of our analysis are consistent with results of the phase 1 study^19^ in demonstrating no evidence of an increased risk of MAO inhibition–related adverse events when ozanimod was administered concomitantly with an SSRI or SNRI, also indicating the lack of in vivo MAO-B inhibition by ozanimod.

In vitro potencies should be considered in the context of in vivo exposures, specifically the unbound or free exposure of the treatment in question.^28^ The likelihood that a treatment will inhibit a target in vivo is generally governed by the ratio of some measure of exposure to potency.^29,30^ For example, differences between in vitro and in vivo MAO inhibition have been demonstrated with linezolid and tedizolid, which are antibiotics with similar weak in vitro MAO inhibition.^29,31^ Linezolid increases blood pressure when coadministered with sympathomimetics, a marker for MAO-B inhibition, while tedizolid does not.^29,31^ These results are consistent with the relationship between the potencies and the free exposures of the molecules.^19,29^ The maximum unbound (free) concentration of linezolid is 21-fold higher than its in vitro IC_50_ for MAO-B inhibition, whereas the maximum unbound concentration of tedizolid is 4.3-fold lower than its in vitro IC_50_.^29^ Using the same analysis for the ozanimod metabolite CC112273, the in vivo maximum unbound concentration of CC112273 is 148-fold lower than its in vitro IC_50_, which is consistent with a lower risk compared with tedizolid for in vivo inhibition of MAO-B.^19,29^ These findings correspond with the results of our analysis, as ozanimod did not increase the risk of TEAEs associated with potential serotonin accumulation regardless of SSRI or SNRI coadministration.

A limitation of this analysis was the relatively small patient population with concomitant ozanimod and SSRI or SNRI use in the pooled UC studies. Additionally, the highly selective patient population included in this analysis from the clinical trials may lack generalizability to the overall population in those who have exclusionary comorbid conditions or factors not represented within the study population. The mean exposure of ozanimod in the pooled UC studies was relatively short, which limits the generalizability of these findings to the general population who receive long-term concomitant ozanimod and SSRI or SNRI. Therefore, continued study in large clinical trials and real-world studies that include elderly patients, various comorbid conditions, and polypharmacy are needed to further evaluate the incidences of TEAEs, especially serious TEAEs, associated with long-term concomitant SSRI or SNRI use with ozanimod.

Despite these limitations, our results from the pooled UC studies and RMS DAYBREAK study did not suggest an increase in TEAEs related to potential serotonin accumulation during the concurrent administration of ozanimod and serotonergic antidepressants. However, it is important to note that the current US prescribing information for ozanimod (June 2024) does not recommend the coadministration of ozanimod with serotonergic agents, and patients should be monitored for hypertension if they are receiving both.^14^

## Data Availability

The Bristol Myers Squibb policy on data sharing may be found at https://www.bms.com/researchers-and-partners/independent-research/data-sharing-request-process.html.
